# A role for BCL2L13 and autophagy in germline purifying selection of mtDNA

**DOI:** 10.1371/journal.pgen.1010573

**Published:** 2023-01-06

**Authors:** Laura S. Kremer, Lyuba V. Bozhilova, Diana Rubalcava-Gracia, Roberta Filograna, Mamta Upadhyay, Camilla Koolmeister, Patrick F. Chinnery, Nils-Göran Larsson

**Affiliations:** 1 Department of Medical Biochemistry and Biophysics, Karolinska Institutet, Stockholm, Sweden; 2 MRC Mitochondrial Biology Unit, School of Clinical Medicine, University of Cambridge, Cambridge, United Kingdom; 3 Department of Clinical Neuroscience, School of Clinical Medicine, University of Cambridge, Cambridge, United Kingdom; University of Cologne, GERMANY

## Abstract

Mammalian mitochondrial DNA (mtDNA) is inherited uniparentally through the female germline without undergoing recombination. This poses a major problem as deleterious mtDNA mutations must be eliminated to avoid a mutational meltdown over generations. At least two mechanisms that can decrease the mutation load during maternal transmission are operational: a stochastic bottleneck for mtDNA transmission from mother to child, and a directed purifying selection against transmission of deleterious mtDNA mutations. However, the molecular mechanisms controlling these processes remain unknown. In this study, we systematically tested whether decreased autophagy contributes to purifying selection by crossing the C5024T mouse model harbouring a single pathogenic heteroplasmic mutation in the tRNA^Ala^ gene of the mtDNA with different autophagy-deficient mouse models, including knockouts of *Parkin*, *Bcl2l13*, *Ulk1*, and *Ulk2*. Our study reveals a statistically robust effect of knockout of *Bcl2l13* on the selection process, and weaker evidence for the effect of *Ulk1* and potentially *Ulk2*, while no statistically significant impact is seen for knockout of *Parkin*. This points at distinctive roles of these players in germline purifying selection. Overall, our approach provides a framework for investigating the roles of other important factors involved in the enigmatic process of purifying selection and guides further investigations for the role of BCL2L13 in the elimination of non-synonymous mutations in protein-coding genes.

## Author summary

We have addressed the role of autophagy on purifying selection of mtDNA by mating different autophagy-deficient mouse models, including knockouts of *Parkin*, *Bcl2l13*, *Ulk1*, and *Ulk2*, to female mice with high levels of the pathogenic tRNA^Ala^ gene mutation. We identified a robust effect of *Bcl2l13* on the selection process, weaker effects of *Ulk1* and *Ulk2*, whereas *Parkin* had no statistically significant impact. Our study thus provides strong experimental evidence for distinctive roles of autophagy in germline purifying selection of mtDNA. Furthermore, the experimental strategy and statistical methods we have developed in the manuscript provide a novel framework for future studies of the enigmatic purifying selection process in mammals.

## Introduction

Mutations in mtDNA are an important cause of mitochondrial disorders where about one in 200 healthy individuals carries a pathogenic mutation in mtDNA and one in 10000 individuals is affected by mtDNA disease [1,2]. Mammalian mtDNA is a circular genome of about 16.6 kb size that can exist in hundreds to thousands of copies per cell. If all mtDNA copies have the same genotype the state is called homoplasmy, whereas heteroplasmy indicates the co-existence of different genotypes in the same cell, e.g., wild-type and mutant mtDNA. The mtDNA is inherited uniparentally through the female germline [3] without undergoing recombination [4]. This poses a major problem, as deleterious mtDNA mutations must be eliminated to avoid a mutational meltdown over generations [5]. Two processes that operate to decrease the mutation load during maternal transmission have been identified, i.e., (i) the genetic bottleneck, which results in the stochastic transmission of only a subset of the pool of mtDNAs present in the mother, and (ii) purifying selection, which prevents transmission of deleterious mtDNA mutations. The mechanisms regulating these processes are unknown, and it is also unclear whether the bottleneck and purifying selection processes are linked to each other.

A possible link between the genetic bottleneck and purifying selection is supported by recent evidence from the C5024T mouse model harbouring a pathogenic C to T substitution within the mitochondrial tRNA^Ala^ gene. In this mouse model, mean heteroplasmy levels are consistent across all tissues [6,7], which simplifies comparisons of heteroplasmy levels between mothers and offspring. When breeding C5024T females, no pups with a heteroplasmy level higher than 80% are obtained [8]. However, increasing mtDNA copy number in the mother via TFAM overexpression shifted the tolerated mutation level in the offspring to 84% [6]. This finding shows that the mtDNA copy number influences purifying selection of mtDNA. The actual mechanism for purifying selection is not yet known, but likely involves a functional test of mtDNA and its ability to sustain normal oxidative phosphorylation (OXPHOS) at the organellar, or cellular level [9]. One appealing hypothesis is that mtDNA mutations impair OXPHOS, which, in turn, leads to depolarization of the mitochondrial inner membrane due to the inability to sustain a normal membrane potential. This decline in OXPHOS function may facilitate subsequent removal of the depolarized mitochondrion by autophagy (also known as mitophagy).

In mammals, several pathways are in place for the removal of mitochondria via autophagy, these might be partially redundant to ensure robustness of the process or allow the removal of mitochondria under specific conditions [10–12]. There are two main pathways that specifically can target mitochondria for degradation by the lysosome involving either ubiquitin- or adapter-dependent marks on the outer mitochondrial membrane. PARKIN plays an important role in the former pathway as it functions as a E3 ubiquitin ligase [13]. Knockout of *Parkin* (also known as *PRKN* or *Park2*) in mice harboring random mtDNA mutations has been suggested to affect the pathogenicity of mtDNA mutations in the striatum and it could possibly also play a role in controlling the inheritance of mtDNA [14]. A representative of the adapter-dependent pathway is BCL2L13, the mammalian homologue of ATG32, which is essential for the autophagic removal of mitochondria in budding yeast [15–17]. Intriguingly, *Bcl2l13* expression can rescue the mitophagy phenotype caused by *Atg32* knockout in yeast [15]. Eventually, most of the mitophagy pathways converge when the mitochondrion is delivered to be engulfed by the autophagosome. Autophagosome formation is initiated by the serine/threonine protein kinase ULK1, the mammalian homologue of ATG1 [18–20]. It was recently reported that knockdown of the *Drosophila melanogaster* homologue of *Ulk1* is necessary for germline selection of mtDNA in the fly [21]. Importantly, ULK2, an ULK1 homologue, shares about 52% protein sequence identity with ULK1 and it is thought that these two proteins act redundantly in some tissues, while in other tissues they might have non-overlapping roles [19,22]. In the current study, we investigated the role of PARKIN, BCL2L13, ULK1, and ULK2 in transmission and purifying selection in the C5024T mouse model.

## Results

### Gene expression levels in the germline

For PARKIN, BCL2L13, ULK1, or ULK2 to contribute to purifying selection, they need to be expressed when selection occurs. Segregation of mtDNA likely occurs during germ cell development, the exact timing is however not known. We therefore checked the expression of the genes encoding these autophagy factors at various stages during germ cell development in publicly available databases and datasets. As these proteins are conserved in mouse and humans, we queried mouse as well as human data. According to the GTEx Portal (GTEx Analysis Release V8 (dbGaP Accession phs000424.v8.p2)) [23] and the Human Protein Atlas [24], PARKIN, BCL2L13, ULK1, and ULK2 are expressed in the adult human ovary. Furthermore, expression of *Bcl2l13*, *Ulk1*, and *Ulk2* transcripts was detected in mouse E12.5 primordial germ cells (PGCs), female germline stem cell (FGSCs), germinal vesicle (GV) oocytes and metaphase II (MII) oocytes, whereas expression of *Parkin* was detected in all stages besides PGCs [25]. As the genes seemed to be readily expressed at various stages of germ cell development in the female germline, they could play a role in the purifying selection of mtDNA.

### Knockout of *Parkin*, *Bcl2l13*, *Ulk1*, or *Ulk2* in C5024T females does not shift the maximal tolerated mutation level in their offspring

**Fig 1:**
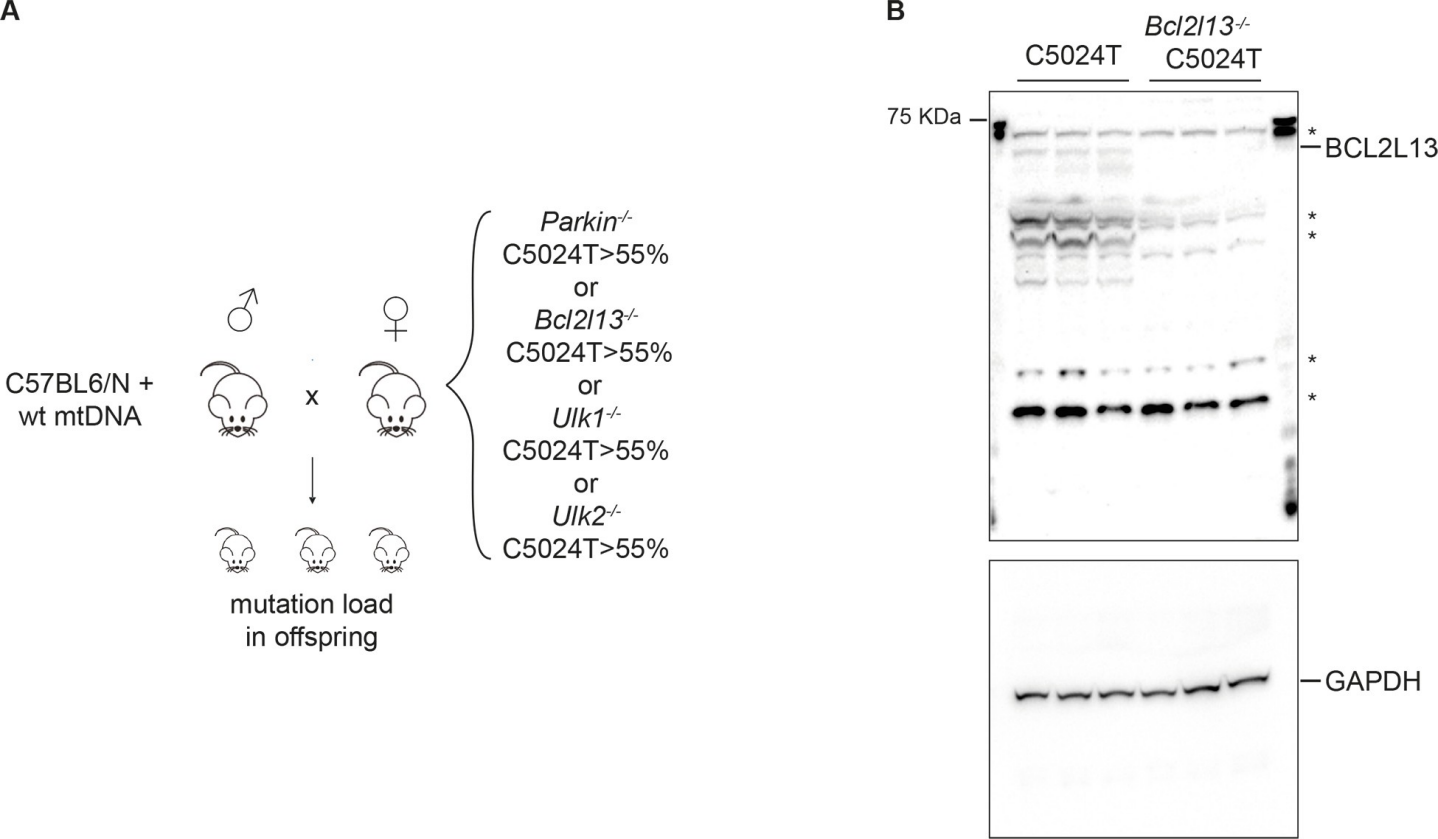
(A) Overview of the experimental setup. Female mice transmitting the pathogenic C5024T mutation in combination with *Parkin*^*-/-*^, *Bcl2l13*^*-/-*^, *Ulk1*^*-/-*^, or *Ulk2*^*-/-*^alleles were crossed to C57BL6/N males with wild-type (wt) mtDNA and the mutation load was measured in the resulting offspring. (B) Western blots analysis of BCL2L13 protein levels in mouse liver (n = 3; * = unspecific bands), levels of GAPDH shown as loading control.

If the removal of mitochondria via autophagy contributes to mtDNA selection, we would expect that knockout of key players in the process would result in a weaker selection. Weaker mtDNA selection, in turn, can manifest in different ways. One possible effect of weaker selection is an increased transmission of mutated mtDNA molecules through the germline, in turn resulting in higher proportions of mutated mtDNA in heteroplasmic mouse pups. This was previously reported to occur as a result of increasing the mtDNA copy number via TFAM overexpression in the C5024T mice [6]. We therefore investigated whether knockout of *Parkin*, *Bcl2l13*, *Ulk1*, or *Ulk2* shifted the maximally tolerated mutation load in the offspring of heteroplasmic C5024T mice. To this end, we generated whole body *Parkin*^-/-^ C5024T, *Bcl2l13*^-/-^ C5024T, *Ulk1*^-/-^ C5024T, and *Ulk2*^-/-^ C5024T females along with *Parkin*^*+/+*^ C5024T and *Bcl2l13*^*+/+*^ C5024T litter mate control females, all on the same mtDNA and nuclear C57BL/6NCrl genetic background (Fig 1A). As the whole body *Bcl2l13* knockout mouse model was newly generated by us, we confirmed that the protein was indeed absent in livers isolated from females after mating (Fig 1B). The *Bcl2l13* knockout mouse model is viable, fertile, and does not develop any obvious phenotype. For each group, we included at least 5 females, all of which had a heteroplasmy level of more than 55% where purifying selection reportedly occurs on transmission [8]. We subsequently crossed these females to C57BL/6NCrl males (Fig 1A) and analyzed the mutation load in the resulting pups.

**Fig 2:**
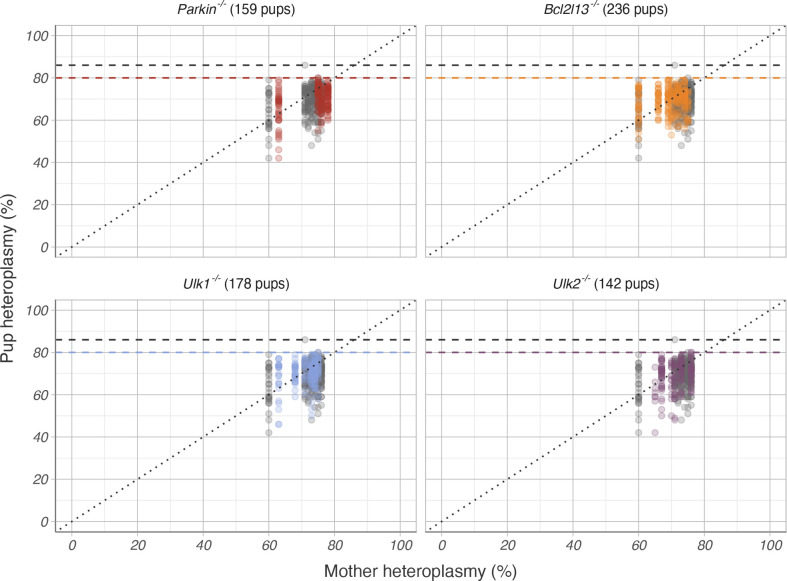
Mother and pup C5024T heteroplasmy across *Parkin*, *Bcl2l13*, *Ulk1* and *Ulk2* knockout groups. Coloured points correspond to mice with autophagy-mediating gene deletion and grey points correspond to mice from both the *Parkin*^*+/+*^ and *Bcl2l13*^*+/+*^ controls (274 pups in total). The dashed lines show the highest observed heteroplasmy level in each group (80% for *Parkin*, *Ulk1 and Ulk2* knockout groups, 79% for the *Bcl2l13* knockout group, and the outlier 86% for *Bcl2l13*^*+/+*^*)*. The highest observed heteroplasmy was comparable across all groups.

In total, we measured the heteroplasmy level from 989 pups born to 40 mothers (Table 1). As we did not observe any difference in heteroplasmy distribution between the *Parkin*^*+/+*^ C5024T and *Bcl2l13*^*+/+*^ C5024T control groups (Kolmogorov–Smirnov test p-value = 0.31) and wanted to keep the animal use to a minimum, we did not generate *Ulk1*^*+/+*^ C5024T or *Ulk2*^*+/+*^ C5024T control groups. Instead, throughout our analysis we used both the *Bcl2l13*^*+/+*^ C5024T and *Parkin*^*+/+*^ C5024T cohorts as controls. It must be noted that out of all samples measured, one sample belonging to the *Bcl2l13*^*+/+*^ C5024T group exceeded the 80% threshold. This turned out to be a technical artefact as remeasurement of the sample revealed a heteroplasmy level below the threshold (1035:417 pup heteroplasmy (%): original measurement: 86; repeated measurement: 71). We did however not want to manually remove this outlier and it is hence included in the analysis. Analysis of the maximally tolerated mutation load in the different groups revealed that neither knockout of *Parkin*, *Bcl2l13*, *Ulk1*, nor *Ulk2* in C5024T mothers had any effect on the maximal tolerated heteroplasmy (Fig 2). This was in contrast to what was observed previously when increasing the mtDNA copy number employing a similar number of breeding pairs and pups [6]. Yet, analyzing the maximally tolerated mutation load is limited as it only takes into account a subset of the samples, namely those pups with the highest heteroplasmy, but does not provide a comprehensive picture on the overall heteroplasmy distribution.

### Knockout of *Bcl2l13*, *Ulk1*, and *Ulk2* affect the heteroplasmy shift from mother to offspring in C5024T mice

**Fig 3:**
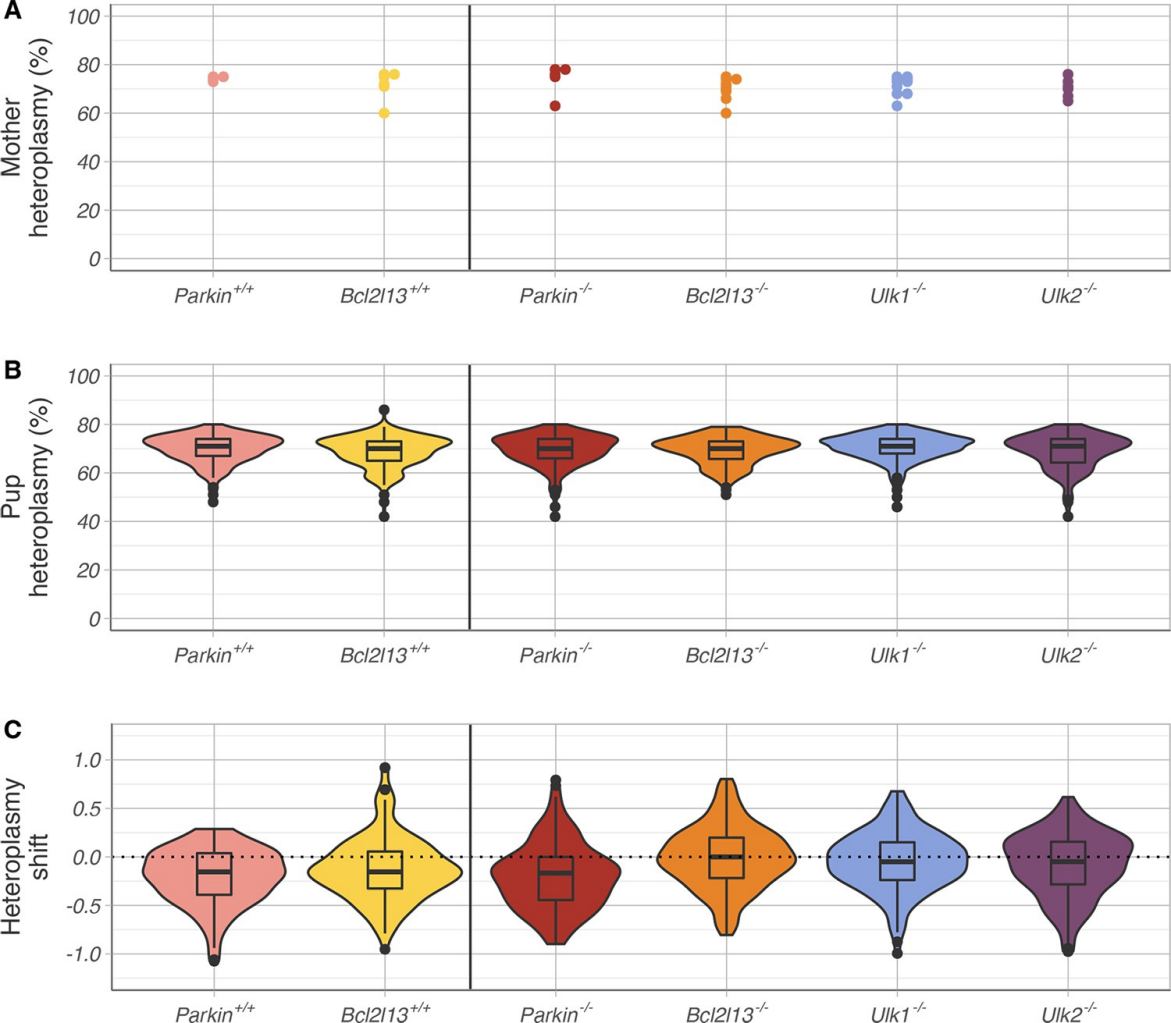
Mother and pup C5024T heteroplasmy across the six groups. (A) High-heteroplasmy (60–78%) mothers were selected in each group. (B) Pups within each group exhibited comparable high heteroplasmy (69.3 ± 6.3% across the cohort). (C) The mean normalised heteroplasmy shift was negative across all groups. The shift was closest to zero for the *Bcl2l13* (-0.01 ± 0.32; orange) and *Ulk1* (-0.04 ± 0.30; blue) knockout groups, indicating that selection may be partially disrupted by the absence of functional BCL2L13 and ULK1.

Weaker mtDNA selection can skew the overall pup heteroplasmy distribution towards higher values, without necessarily changing the maximal heteroplasmy. Pup heteroplasmy is not independent of maternal heteroplasmy [7], and therefore differences in maternal heteroplasmy across groups may confound any comparative analysis. As it is not possible to strategically breed mothers with the exact same heteroplasmy level for the different groups (Fig 3A), we instead calculated a mother-to-offspring normalized heteroplasmy shift from the observed heteroplasmy measurements which accounts for this confounder in the analysis (Fig 3B and 3C) [7,26]. In the absence of selection pressure, the mean heteroplasmy shift is zero [27], but if the mean heteroplasmy shift is negative, this is indicative of purifying selection. In agreement with previously reported results [7], purifying selection was present in high-heteroplasmy C5024T mothers across both *Parkin*^*+/+*^ C5024T and *Bcl2l13*^*+/+*^ C5024T control groups, where the mean heteroplasmy shift was -0.20 ± 0.29 and -0.12 ± 0.33 (mean ± st.dev.) respectively (Table 1 and Fig 3C). A similar negative shift was also observed in the *Parkin* knockout group (-0.20 ± 0.34). However, the shift in the *Bcl2l13* knockout group was closer to zero (-0.01 ± 0.32), and shifts in the *Ulk1* and *Ulk2* knockouts were higher than in both controls (-0.04 ± 0.30 and -0.08 ± 0.33, respectively). Of note, none of the gene knockouts had any obvious effect on the normalized heteroplasmy variance of the pups (S1 Fig).

**Table 1:** Mothers and pups in each genotype group. Pup heteroplasmy, and mother-to-pup heteroplasmy difference and normalised heteroplasmy shift are given as mean ± st.dev.

Nuclear genotype group	# mothers	# pups	Pup heteroplasmy (%)	Heteroplasmy difference from mother (%)	Normalised heteroplasmy shift
***Parkin:+/+***	4	122	69.73±6.04	-4.39±6.18	-0.20±0.29
***Bcl2l13:+/+***	6	152	68.70±6.61	-2.66±7.00	-0.12±0.33
***Parkin:-/-***	5	159	69.40±6.57	-4.12±7.07	-0.20±0.34
***Bcl2l13:-/-***	9	236	69.19±5.76	-0.28±6.94	-0.01±0.32
***Ulk1:-/-***	10	178	70.19±6.02	-1.03±6.52	-0.04±0.30
***Ulk2:-/-***	6	142	68.84±7.11	-2.02±7.12	-0.08±0.33

This initial exploratory analysis suggests that knockouts of *Bcl2l13*, *Ulk1* and *Ulk2* have effect on the mean heteroplasmy shift, and therefore on purifying selection. Wilcoxon’s rank tests, with Bonferroni multiple-testing correction show that the shifts in the *Parkin*^*+/+*^, *Bcl2l13*^+/+^, and *Parkin*^-/-^ groups were significantly different from zero (p-value < 0.001 in all cases), while the shifts in *Bcl2l13*^-/-^, *Ulk1*^-/-^, and *Ulk2*^-/-^ were not (p-values 0.678, 0.095, and 0.015 respectively). However, in the data, mothers have multiple pups (24.73 ± 9.76), and therefore each mother-offspring observation is not independent, violating standard statistical hypothesis test assumptions. Indeed, heteroplasmy shift distributions varied by mother, albeit not to the extent that a single mother would have an outsized effect on the shift distribution in the whole cohort (S2 Fig). Nevertheless, any measurement errors on the maternal heteroplasmy levels could propagate across tens of observations and bias the results.

**Fig 4:**
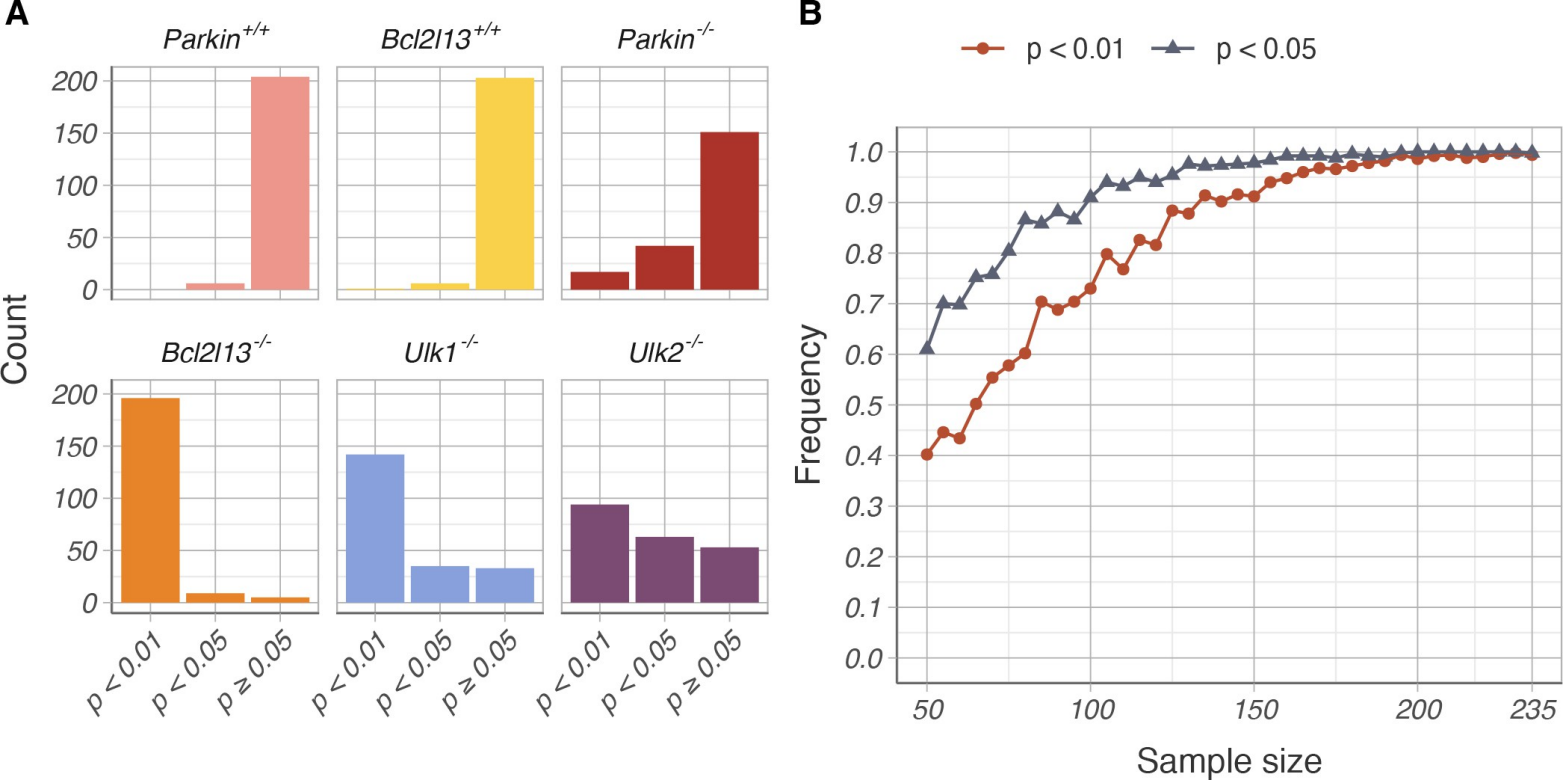
Robustness analysis of results. (A) Proportion of bootstrap p-values showing a significant difference between subsampled control data and the six experimental groups. As expected, *Parkin* (pink) and *Bcl2l13* (yellow) wildtype groups were not significantly different to most subsampled controls. The *Parkin* knockout (red) also had no significant effect on the C5024T heteroplasmy shift. The *Bcl2l13* knockout group (orange) was consistently significantly different from subsampled controls. Tests for the *Ulk1* (blue) and *Ulk2* (purple) knockouts showed more variable results, with the effect of *Ulk1* knockout being significant in over half of samples. (B) Subsampling of the *Bcl2l13* knockout data and controls shows the effect is consistently observed at lower sample sizes.

In order to robustly test whether the effect of knockout of *Bcl213*, *Ulk1*, and *Ulk2* on the normalized heteroplasmy shift was statistically significant, we performed Kolmogorov–Smirnov tests comparing gene knockout groups to subsamples of the control data. Testing the larger of the control groups, *Bcl2l13*^*+/+*^ C5024T (six mothers, 152 pups), against all other groups indicated that only the *Bcl2l13* and *Ulk1* knockouts resulted in significantly different distribution of heteroplasmy shifts (p-value < 0.001 and 0.005 respectively). To ascertain that our results were not because we restricted the control data to the six *Bcl2l13*^*+/+*^ C5024T mothers, we further generated all 210 possible subsampled control datasets, consisting of any six mothers and all their respective offspring from the *Bcl2l13*^*+/+*^ C5024T and *Parkin*^*+/+*^ C5024T groups. For example, one such subsampled control could consist of all four mothers in the *Parkin*^*+/+*^ C5024T group and two out of the six mothers in the *Bcl2l13*^+/+^ C5024T group. Another subsampled control could consist of one mother from the *Parkin*^*+/+*^ C5024T group and five mothers from the *Bcl2l13*^*+/+*^ C5024T group. We subsequently performed Kolmogorov–Smirnov tests for the *Parkin*^*+/+*^ C5024T and *Bcl2l13*^*+/+*^ C5024T control groups and each knockout group against each of the 210 subsampled controls. We measured how frequently the genotype groups were significantly different to the subsampled controls (p-value < 0.008 after Bonferroni multiple-testing correction). Under the null hypothesis that heteroplasmy shifts are identically distributed between control and knockout groups, the median p-value would be 0.50. The proportion of subsampled control tests resulting in p-values below 1% and below 5% for each genotype group are depicted in Fig 4A. As expected, the *Parkin*^*+/+*^ C5024T and *Bcl2l13*^*+/+*^ C5024T control groups did not significantly differ from any of the 210 subsampled controls (see also Fig 4A, left and middle upper panel). Significant differences of the *Parkin* knockout group were rare (14 out of 210 tests, or 6.67% of tests), indicating the knockout is indeed unlikely to affect the heteroplasmy shift (Fig 4A, right upper panel). In contrast, the *Bcl2l13* knockout group was significantly different from the vast majority of subsampled controls (195 out of 210 tests, or 92.86% of all tests), providing robust evidence of the *Bcl2l13* effect on purifying selection (Fig 4A, left lower panel). For the *Ulk1* group, over half of tests showed significant difference (138 out of 210 tests, or 65.71% of all tests), with median p-value = 0.002 below the significance threshold (Fig 4A, middle lower panel). Results for the *Ulk2* group were similar to the results for *Ulk1* but weaker with less than half (90 out of 210 tests, or 42.86% of all tests) showing a statistically significant difference, with a median p-value = 0.013 above the threshold (see also Fig 4A, right lower panel). To ensure that the results for the *ULK1* and *ULK2* groups were not due to a residual carry-over of their original C57BL/6J genetic background, we genotyped all mothers for the *NNT* mutation as marker thereof [28]. Indeed, four mothers from the *Ulk1* group still carried the *NNT* mutation, referred to as NNT mothers. Importantly, the *NNT* mutation was heterozygous in the NNT mothers, meaning that the NNT mothers have a functional copy of *NNT*. Accordingly, there was no difference in heteroplasmy distributions between wildtype and NNT mothers (see S3A Fig) and excluding the NNT mothers from the subsampling analysis yielded comparable results to using the full dataset (see S3B Fig). We therefore conclude that our results are not due to any differences in the nuclear genetic background.

Finally, we wanted to ascertain whether the effect observed for the knockout of *Bcl2l13* was not simply due to the bigger sample size in this group (236 pups compared to 122–159 pups in other groups). We therefore repeated the Kolmogorov–Smirnov test comparing *Bcl2l13* ^-/-^ C5024T to controls by resampling both knockout and control data with replacement. Knockout and control subsamples of different sizes were repeatedly generated, and the fraction of significant differences for each sample size was calculated (Fig 4B). For sample size n = 120, the effect of *Bcl2l13* knockout is already reliably detected in 81.6% of replicates at the 1% significance level. Therefore, we concluded that for all our knockout groups our sample size was sufficient to detect a statistically significant difference in the distribution of normalized heteroplasmy shift.

In summary, our study reveals a statistically robust effect of knockout of *Bcl2l13* on the selection process, and weaker evidence for an effect of *Ulk1* and potentially also for *Ulk2*, while no statistically significant impact is seen for knockout of *Parkin*.

## Discussion

Inherited mutations of mtDNA are a major cause of rare mitochondrial disorders and are also implicated in the aging process itself as well as in common degenerative diseases and cancer [1,29–31]. Importantly, the incomplete understanding of the very different genetics of mtDNA disorders largely prevents a similar progress as has been made in diagnostics and genetic counseling for disease caused by mutations in nuclear genes. A thorough understanding of mtDNA transmission is not only of utmost importance to provide families at risk of transmitting mtDNA disorders with advice about the recurrence risks, but understanding the mechanisms may also provide clues to prevent disease recurrence and limit the impact of mtDNA mutations on common disorders.

Although the first steps towards manipulating heteroplasmy transmission have been made by experimentally deciphering the role of increasing mtDNA copy number on the propagation of the C5024T mutation [6], modulating the mtDNA copy number in human germ cells is not currently possible, making it important to explore other strategies. One of these approaches could be to manipulate bulk or selective autophagy of mitochondria. To explore the connection between autophagy and mtDNA purification, we systematically introduced knockout alleles for several players involved in autophagy in the C5024T mouse model and performed a comprehensive analysis of the possible effects on purifying selection. Crucially, our analysis takes into account the relatively low number of mothers in the data, and the fact that mother-to-offspring observations are not independent. Our study reveals a robust effect of knockout of *Bcl2l13* on the selection process, and weaker evidence for an effect of *Ulk1* and potentially also for *Ulk2*, while no statistically significant effect was seen for knockout of *Parkin*. This indicates distinctive roles of the different players in purifying selection and is in line with recent findings on mtDNA inheritance in the fly and somatic mtDNA segregation in mice [32,33]. BCL2L13 can rescue knockout of *Atg32* and restore mitophagy in budding yeast [15], suggesting that it has a role for mitophagy in mammals. However, the precise function of BCL2L13 is still unknown and further studies are needed to determine whether enhancing BCL2L13 function can counteract transmission of mutant mtDNA.

Although our study is an important step forward in understanding purifying selection of mtDNA in the female mammalian germline, it has several limitations. First, we only investigated one mutation (C5024T), which is relatively well tolerated in the mouse. The impact of knocking out key autophagy proteins could be different for other mutations, especially for other mutations leading to more severe phenotypes. Secondly, as mentioned above, several pathways exist for the removal of mitochondria via autophagy. Unexplored pathways, mitigated by other players in the adapter-dependent pathway for example, may be fruitful avenues of further investigation [11]. Thirdly, for technical reasons we began our exploration by knocking out key autophagy proteins in order to show ‘proof or principle’ that mitophagy is important in heteroplasmy transmission. It will therefore be important to explore ways of enhancing autophagy to see if this suppresses the transmission of mutant mtDNA. Fourthly, although ideally it would be helpful to confirm our findings with littermate *Ulk1*^*+/+*^ C5024T and *Ulk2*^*+/+*^ C5024T control groups, given the lack of significant difference between *Parkin*^*+/+*^ C5024T and *Bcl2l13*^*+/+*^ C5024T control groups, and the need to minimise unnecessary animal use, it would be ethically contentious to repeat these observations to confirm a negative result. Finally, functional redundancy may mean that the simultaneous knockout of several proteins will be required to sufficiently disrupt mitophagy to modulate heteroplasmy transmission. In keeping with this, *Ulk1* knockout and *Ulk2* knockout mice appear to be healthy, but mice with a double knockout of *Ulk1* and *Ulk2* die shortly after birth, showing that at least one of these pathways must be present to allow postnatal development [34]. Functional redundancy between ULK1 and ULK2 may also explain why individually they exhibit weaker effects on heteroplasmy transmission. Further experiments are therefore needed to better elucidate the role of autophagy as a contributor to effective selection of mtDNA in the female germline. Moreover, the selective removal of dysfunctional mitochondria is just one of several possible mechanisms governing selection. Other possibilities include processes involved in the preferential propagation of mutant mtDNA, pathways independent of the mitochondrial membrane potential, positive selection of healthy mitochondria, and mechanisms acting on the cellular level rather than on the organelle level [9,35]. These mechanisms are not mutually exclusive, making it possible that several act at multiple levels to ensure faithful propagation of functional mtDNA to the next generation. This would make evolutionary sense given the potentially catastrophic effects of the relentless accumulation of mtDNA mutations down the female germ line, leading to ‘mutational meltdown’ and extinction of the species (Mullers ratchet) [36].

Overall, our findings establish a robust role for BCL2L13 in purifying selection of mtDNA in the female germline, and provide both an entry point to determine the effects of other autophagy modulators, and a methodological approach to further dissect molecular mechanisms for this essential and incompletely understood phenomenon.

## Materials and methods

### Ethics statement

The study was approved by the Stockholm ethical committee (Stockholms djurförsöksetiska nämnd) under the ethical permit 2001–2018 and performed in accordance with the guidelines of the Federation of European Laboratory Animal Science Associations (FELASA).

### Mice

Whole body *Bcl2l13* knockout mice were generated by the Karolinska Center for Transgene Technologies using the CrisprCAS9 system. Two guide RNAs (sgRNA1: TCCTCTACGACTGCGTCTCT, sgRNA2: CTGCAGTCCATGCCAGCGGA) targeting exon 2 and 5 of *Bcl2l13*, respectively, were injected alongside Cas9 protein into C57BL/6NCrl zygotes twice. Out of the 38 animals born, three animals carried deletions around the guide RNA targeting exon 5. Out of these 3 founder animals, the animal carrying a 108 bp deletion (g.34512_34619del, NCBI Gene ID: 94044; g.120847678_120847785del, NCBI Reference Sequence: NC_000072.6) was chosen to establish the *Bcl2l13* knockout mouse line. The C5024T mice were generated previously [8]. Whole body *Parkin* knockout mice were obtained by crossing mice with *Parkin* exon 7 flanked by loxP-sites [37] to β-actin-cre transgenic mice [38] followed by backcrossing to C57BL/6NCrl mice (Charles River, Germany). Whole body *Ulk1* and *Ulk2* knockout mice were created by mating *Ulk1*^FL^ and *Ulk*2^FL^ mice (The Jackson Laboratory, stock number 017976 and 017977, respectively) to β-actin-cre transgenic mice [38] and subsequent backcrossing for 5 generations to C57BL/6NCrl mice (Charles River, Germany). Animals were housed in a 12-hours light/dark cycle in standard ventilated cages and fed *ad libitum* with a normal chow diet.

### Tissue isolation

Animals were euthanized by CO_2_ followed by cervical dislocation. Liver was collected immediately, washed with phosphate-buffered saline (PBS), snap-frozen in liquid nitrogen, and stored at -80°C.

### Protein extractions and immunodetections

Livers were ground in liquid nitrogen using mortar and pestle. Powder was resuspended in 500 μL of lysis buffer (25mM HEPES, pH 7.5; 5mM MgCl_2_; 0.5mM EDTA; 1x EDTA-free protease inhibitor cocktail (Roche); 1% NP-40 and 140 mM NaCl) and homogenized for 15 sec with the T 10 basic ULTRA-TURRAX system. Samples were centrifuged at 13000 rpm for 10 min at 4°C and supernatants were collected. Protein concentration was determined with the BCA protein assay kit (Pierce) and 75 μg of protein was resolved by electrophoresis using 12% Bis-Tris Plus NuPAGE gels (Invitrogen) and transferred to PVDF membranes (iBlot 2 system (Invitrogen)). BCL2L13 was detected (Proteintech 16612-I-ap) and GAPDH (ab8245) was used as a loading control.

### Quantification of the C5024T mutation level

The C5024T mutation level was determined as described previously from DNA isolated from ear clips around the timing of weaning [8]. Briefly, a 178-base pair fragment spanning the C5024T site was amplified using the primers 5′Biotin TTCCACCCTAGCTATCATAAGC (forward) and GTAGGTTTAATTCCTGCCAATCT (reverse). PCR products were purified using PyroMark binding buffer (Qiagen) and 1 μl Streptavidin Sepharose TM high-performance beads (GE Healthcare) and denatured with a Pyromark Q24 vacuum workstation (Qiagen). Sequencing was performed using the sequencing primer TGTAGGATGAAGTCTTACA and PyroMark Gold Q24 Reagents (Qiagen) according to the manufacturer’s instructions on a PyroMark Q24 pyrosequencer (Qiagen).

### Statistical analysis

Data analysis and figure generation were performed in R v.4.2.1 using standard libraries.

Mother-to-pup normalized heteroplasmy shift was calculated as

$$shift=\ln\left(\frac{H_{pup}}{1-H_{pup}}\right)-\ln\left(\frac{H_{mother}}{1-H_{mother}}\right),$$

where *H_pup_* and *H_mother_* are the heteroplasmy fractions of the pup and the mother, respectively [26].

Normalised heteroplasmy variance for each mother was calculated as

$$V_{mother}^{\prime }=\frac{var(H_{pups})}{mean(H_{pups})(1-mean(H_{pups}))},$$

where *H_pups_* are the heteroplasmy fractions of all pups born to that mother, and *mean*(·) and *var*(·) denote the sample mean and sample variance respectively.

Across this study, Wilcoxon’s rank tests and Kolmogorov–Smirnov tests on the heteroplasmy shift were carried out at the 5% significance level. A Bonferroni multiple-testing correction was applied with *n* = 6 when testing all genotype groups simultaneously, and *n* = 5 when testing the *Bcl2l13*^*+/+*^ C5024T control against the other five genotype groups.

When investigating sample size effects, control and *Bcl2l13* knockout data of equal size were generated by sampling 500-fold with replacement. Sample sizes considered ranged from *n* = 50 to *n* = 235 at intervals of five.

## Supporting information

S1 Fig Normalized C5024T heteroplasmy variance of pups, split by mother. While a range of normalized variances was observed (0.003 to 0.041), gene knockouts did not appear to have an effect on the pup heteroplasmy variance.
(TIF)

S2 Fig C5024T heteroplasmy shift by mother. Solid lines represent the mean shift across each genotype group, and dotted lines represent the mean ± 1.5 st.dev. In every genotype group, mother-to-pup heteroplasmy shift varied by mother. Nevertheless, no mothers had a significant proportion of outlier shifts, and no individual mother had an outsized effect on the overall group shift distribution.
(TIF)

S3 Fig C5024T heteroplasmy shift analysis of pups born to *Ulk1_-/-_* C5024T mothers not carrying the *NNT* mutation (6 mothers, 111 pups). (A) Heteroplasmy shift distributions between *NNT* wildtype mothers (WT) and mothers carrying the *NNT* mutation (NNT) were comparable (Kolmogorov-Smirnov p-value = 0.194). (B) When the subsampling analysis was repeated excluding the mothers carrying the *NNT* mutation, over half of tests (127 out of 210, or 60.48%) remained significant, with median p-value 0.005 below the significance threshold.
(TIF)

S1 Data The numerical data underyling the results of this study. (XLSX)
