# Supplementary figures and images for: A role for BCL2L13 and autophagy in germline purifying selection of mtDNA
Source: PLoS Genet. 2023 Jan 6;19(1):e1010573. doi: 10.1371/journal.pgen.1010573 (PMC9851501; doi:10.1371/journal.pgen.1010573)

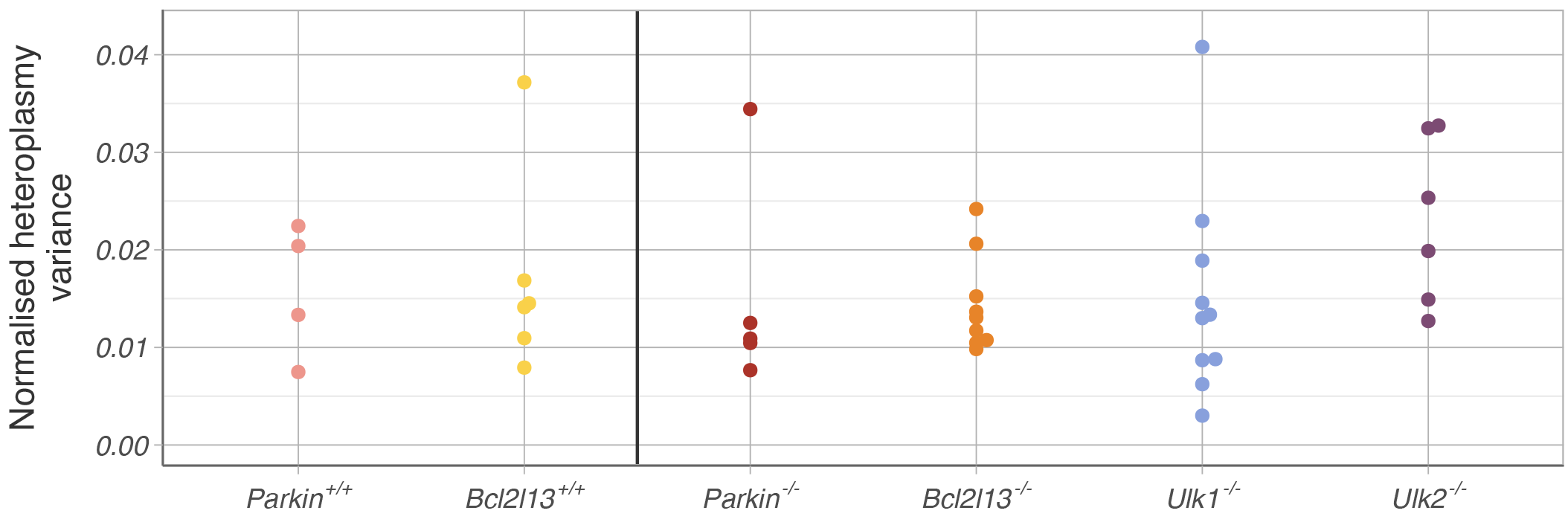

Supplement: S1 Fig — While a range of normalized variances was observed (0.003 to 0.041), gene knockouts did not appear to have an effect on the pup heteroplasmy variance. (TIF) [file pgen.1010573.s001.tif]

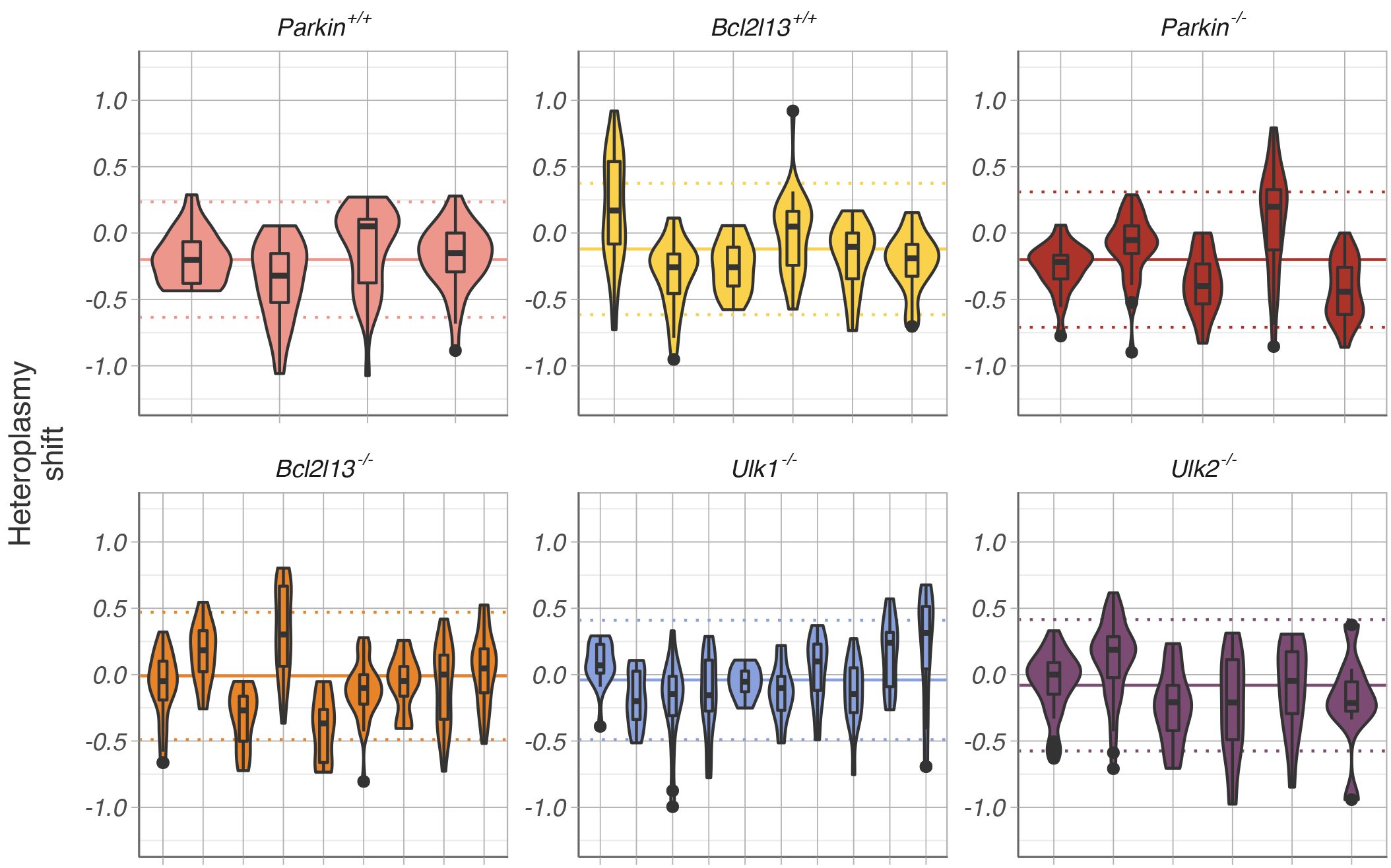

Supplement: S2 Fig — Solid lines represent the mean shift across each genotype group, and dotted lines represent the mean ± 1.5 st.dev. In every genotype group, mother-to-pup heteroplasmy shift varied by mother. Nevertheless, no mothers had a significant proportion of outlier shifts, and no individual mother had an outsized effect on the overall group shift distribution. (TIF) [file pgen.1010573.s002.tif]

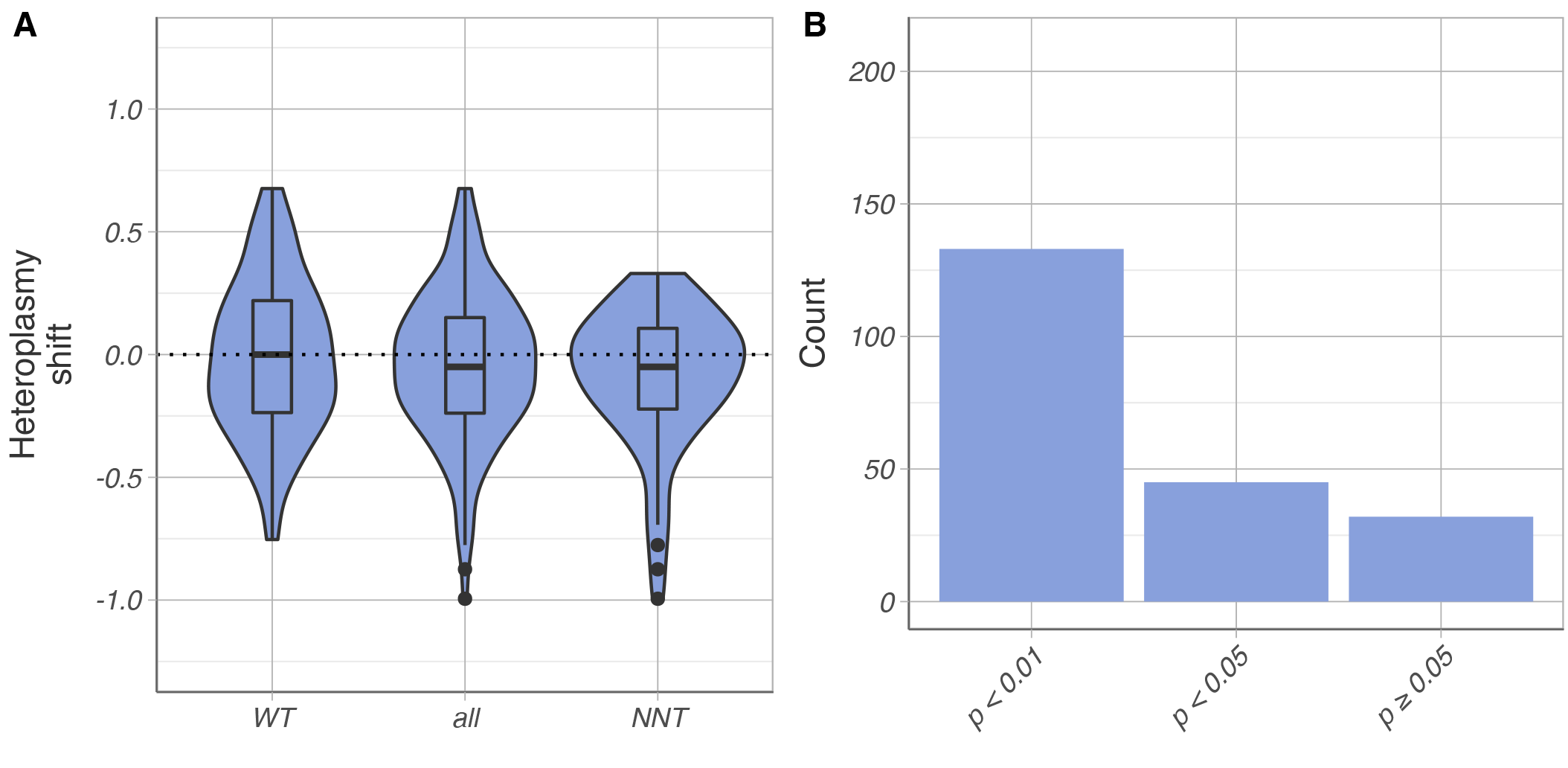

Supplement: S3 Fig — (A) Heteroplasmy shift distributions between NNT wildtype mothers (WT) and mothers carrying the NNT mutation (NNT) were comparable (Kolmogorov-Smirnov p-value = 0.194). (B) When the subsampling analysis was repeated excluding the mothers carrying the NNT mutation, over half of tests (127 out of 210, or 60.48%) remained significant, with median p-value 0.005 below the significance threshold. (TIF) [file pgen.1010573.s003.tif]
